# Hepatic arterial infusion chemotherapy and trastuzumab in gastric cancer with liver metastases: a case report

**DOI:** 10.3389/fonc.2023.1283274

**Published:** 2023-12-21

**Authors:** Hui-qin Li, Qin Wang, Liu-yan Zhang, Jia-yin Li, Ying-jie Wang, Li Wei, Li-ge Yao

**Affiliations:** Department of Oncology, The Third People’s Hospital of Zhengzhou, Zhengzhou, China

**Keywords:** gastric cancer, liver metastases, hepatic arterial infusion chemotherapy, trastuzumab, human epidermal growth factor receptor 2

## Abstract

**Background:**

Gastric cancer is a common cancer worldwide and is responsible for over one million new cases in 2020 and an estimated 769,000 deaths, ranking fifth for incidence and fourth for mortality globally. Incidence rates are highest in Eastern Asia and Eastern Europe. Gastric cancer is highly heterogeneous and progresses rapidly. The prognosis of gastric cancer with liver metastases is poor, and clinical treatment remains challenging. Human epidermal growth factor receptor 2 (HER2) positivity is correlated to a bad prognosis for gastric cancer. Trastuzumab combined with systemic chemotherapy is the preferred treatment for HER2-positive advanced gastric cancer. However, intravenous chemotherapy has severe systemic toxicity, which reduces the local drug concentration and tumor uptake rate, and the effect is unsatisfactory.

**Case summary:**

We reported a 66-year-old patient with HER2-positive advanced gastric cancer with jaundice due to multiple liver metastases, after 6 cycles of trastuzumab combined with hepatic arterial infusion chemotherapy (HAIC), the tumor retracted significantly, the jaundice subsided, and the patient recovered well. The patient achieved disease control with an intensive regimen followed by less toxic maintenance therapy. Trastuzumab combined with capecitabine maintenance therapy followed up for more than 16 months.

**Conclusion:**

HAIC plus trastuzumab may be a tolerable treatment option for patients with severe liver metastases from HER2-positive gastric cancer to achieve local control and prolong survival.

## Introduction

Gastric cancer (GC) is the fifth most common malignancy in the world, with a high mortality rate ranking fourth (1). The liver is one of the most frequently metastasized organs in advanced gastric cancer, with approximately 30% of patients experiencing blood metastasis through portal vein circulation (2–5). Usually, the first-line chemotherapy regimen is based on fluorouracil drugs and combined with platinum and/or paclitaxel drugs to form a two or three-drug chemotherapy regimen. Although the three-drug regimen DCF (Docetaxel, Cisplatin, 5-FU) met the study endpoint in the phase III study, its high toxicity limited its clinical use. In China, a combination of fluorouracil and platinum drugs is recommended. Due to better patient tolerance and the current status of real-world clinical treatment applications in China, platinum drugs are more recommended as oxaliplatin (6–15). Approximately 10-22% of GC patients have HER2 overexpression. The ToGA trial suggests that trastuzumab and chemotherapy prolonged overall survival (OS) for 2.7 months and improved objective response rate (ORR) in gastric cancer patients with HER2-positive compared with chemotherapy alone, therefore trastuzumab has been added to conventional chemotherapy in patients with advanced gastric cancer (16). The 5-year survival rate of gastric cancer with multiple liver metastases (GCLM) was as low as 6% to 13.1% (17). Meanwhile, intravenous chemotherapy has severe systemic toxicity, which reduces the local drug concentration and tumor uptake rate, and the effect is unsatisfactory.

HAIC is a kind of interventional therapy that can increase the concentration of liver tumor drugs and improve the anti-tumor effect by injecting chemotherapy drugs through the hepatic artery. By using the trans-arterial route of administration, drug concentrations in the liver metastases can be significantly increased compared with the intravenous route, resulting in a 3- to 5-fold increase in the response rate of 5-FU and oxaliplatin (18). The feasibility, safety, and efficacy of hepatic artery infusion chemotherapy have been confirmed in colorectal cancer patients (19, 20). Therefore, researchers have turned their attention to the application of HAIC in GCLM. Wang et al. reviewed five GCLM patients who received HAI oxaliplatin and S-1 oral treatment, the objective response rate was 40%, the disease control rate was 80%, and HAI chemotherapy had better local control of liver metastasis, with a median PFS of 8.8 months (21). Ojima et al. retrospectively evaluated the efficacy of HAIC for synchronous GCLM, ORR of HAIC was 83%, there were no serious side effects, and the median survival time (MST) of the HAIC group was 19.2 months (22). In the currently published studies on the treatment of gastric cancer with liver metastases (GCLM), there are no reports of HAIC combined with trastuzumab. We report a HER2-positive GCLM patient who received this combination approach in first-line treatment, which resulted in tumor control, improved quality of life, and improved prognosis.

## Case presentation

**Figure 1:**
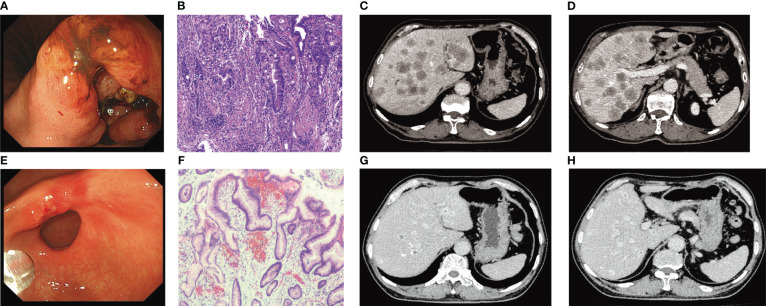
**(A)** Gastroscopy before treatment. **(B)** Pathological findings of gastroscopic biopsy (HE×200) (suggesting moderately differentiated adenocarcinoma). **(C, D)** Abdominal CT before treatment. **(E)** Results of gastroscopy examination after 6 cycles of treatment (disappearance of lesions before treatment). **(F)** Pathological examination results after 6 cycles of treatment (HE×200). **(G, H)** Evaluation of Partial response (PR) after 6 cycles of trastuzumab combined with HAIC treatment.

A 66-year-old male had abdominal distension, sour regurgitation, and heartburn, aggravated by full eating and spicy food for 1 month, but did not receive treatment for his symptoms, came to our hospital in April 2022. The patient was previously a taxi driver with an irregular diet and a history of Helicobacter pylori infection and did not receive eradication treatment. He had a history of hypertension, and arrhythmia, and taken oral valsartan, hydrochlorothiazide, and metoprolol medications, and no family history related to tumors. His Eastern Cooperative Oncology Group (ECOG) performance status (PS) was 2. Physical examination: no palpable enlargement of superficial lymph nodes throughout the body, no tenderness or rebound tenderness in the abdomen, and no palpation under the hepatic subcostals. His laboratory findings, including AST, 100 U/L; ALT, 127U/L; and total bilirubin (T. Bil), 75.4μmol/L, indirect bilirubin (I. Bil), 55.1μmol/L, suggested severe hepatic injury. Laboratory tests suggested that his kidney function was normal. Tumor marker detection indicated CEA>2000ng/ml, CA50 105.66U/ml, CA199>1000U/ml, CA72-4 251.88U/ml, and CA24-2 57.36U/ml. Cardiac ultrasound indicated that the left ventricular ejection fraction (LVEF) was normal. Gastroscopy revealed an ulcerating mass in lesser gastric curvature near the gastric antrum (**Figure 1A**). Gastroscopic biopsies showed moderately differentiated adenocarcinoma, as shown in **Figure 1B**. Immunohistochemistry assay showed HER2 positive staining, microsatellite stability, PD-L1 CPS 0, and Ki-67 (80%). Abdominal computed tomography (CT) revealed a lump in the gastric antrum, accompanied by enlarged lymph nodes in the hepatogastric space; Multiple enlarged nodules were observed in the liver, as shown in **Figures 1C, D**. Thoracic CT showed no signs of lung metastasis. Bone scintigraphy showed no evidence of bone metastasis. Head magnetic resonance imaging (MRI) indicated no signs of brain metastasis.

**Figure 2:**
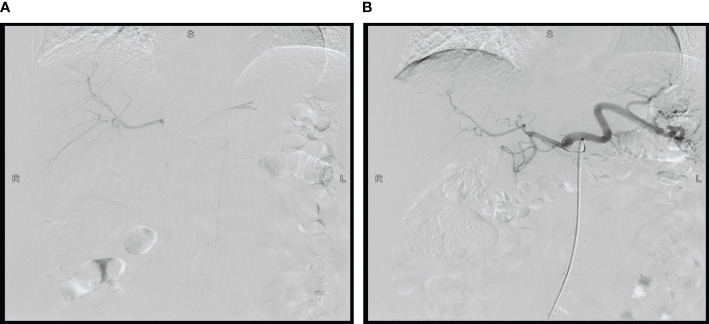
Hepatic arterial infusion chemotherapy **(A, B)**.

Considering the large tumor burden and liver metastases, we devised a therapeutic regimen consisting of targeted drugs and HAIC. The treatment regimen included intravenous Trastuzumab, hepatic arterial infusion of oxaliplatin, and fluorouracil, and the treatment was repeated after one cycle (21 days). The initial load dose of trastuzumab was 8 mg/kg, followed by 6 mg/kg every 3 weeks. During the regular treatment of trastuzumab, we monitored the patient’s cardiac enzymes, electrocardiogram, and cardiac color ultrasound, and did not reduce or discontinue the drug due to adverse reactions. For HAIC, after ultrasonic positioning, the Seldinger method was used to puncture the femoral artery, and after successful insertion of 5F arterial sheath, the catheter guide wire was sent along the sheath, selective insertion of abdominal trunk arteriography, the catheter with a drug delivery device and insert the tip into the common hepatic artery after imaging confirmation. Oxaliplatin (80 mg/m^2^ for 2 hours) and fluorouracil (2,600 mg/m^2^ for 72 hours) were administered through the port catheter system (**Figure 2**). After one cycle of HAIC combined with intravenous trastuzumab, jaundice quickly subsided and total bilirubin decreased to normal. The level of tumor markers significantly decreased. Partial response was observed after 1 month. After receiving six cycles of treatment, the patient achieved maximum efficacy (**Figures 1G, H**). Gastroscopy showed no mass and ulcer (**Figure 1E**); Pathological biopsy showed no tumor cells (**Figure 1F**). The patient’s ECOG score returned to a level of 1. The significant adverse reactions during HAIC were fatigue, neuropathy, and mild nausea, which improved after symptomatic treatment, with no grade 3-4 adverse effects.

**Figure 3:**
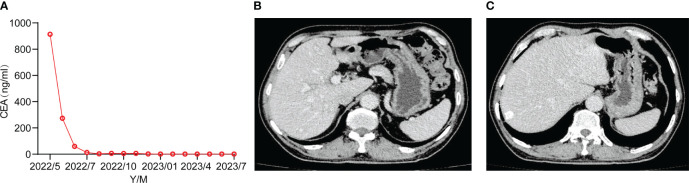
**(A)** Tumor markers returned to normal. **(B, C)** Follow-up CT in July 2023 assessed the stability.

To improve the patient’s quality of life and prolong his survival time, maintenance therapy is recommended. The optimal mode of maintenance therapy in the first-line advanced gastric cancer has not been established. Targeted therapy combined with monochemotherapy as a maintenance regimen may be the choice in clinical practice. We recommend the combination of intravenous trastuzumab and capecitabine for maintenance treatment, with a specific dose of 6mg/Kg of trastuzumab; Capecitabine 1000mg/m^2^, taken orally for 2 weeks, stopped for 1 week, every 3 weeks. During the oral administration of capecitabine, the patient experienced nausea, acid reflux, and neuropathy, and capecitabine was reduced by 20%. At present, the tumor markers of the patient are normal (**Figure 3A**), and the imaging assessment is stable (**Figures 3B, C**). In the follow-up maintenance treatment of trastuzumab combined with capecitabine, the patients have been followed up for more than 16 months.

## Discussion

The treatment of liver metastases from gastric cancer remains debatable. The median overall survival of intravenous fluorouracil combined with platinum chemotherapy is 10.5-11.6 months. With the development of precision medicine, molecular-targeted therapy has become the main treatment method for gastric cancer. HER2 plays an important role in the biological behavior and pathogenesis of advanced GC and is also an important target for the systemic treatment of advanced GC at present (23). About 15%-20% of patients have abnormal activation or overexpression of HER2, which is significantly associated with poor prognosis of patients (24). Trastuzumab plus systemic chemotherapy has become the standard first-line treatment for HER2-positive advanced gastric cancer, with a median overall survival of 13.8 months. However, for gastric cancer patients with jaundice due to extensive liver metastases, the effect of systemic chemotherapy is unsatisfactory, while the patients often experience serious adverse reactions, their quality of life is affected, the compliance decreases, and their prognosis is poor (25). Since one of the key factors determining prognosis is liver metastases, local control is considered to be very important (22).

HAIC is a treatment that continuously infuses chemotherapy drugs into tumors through hepatic arteries. Hepatic metastases derive their blood supply mainly from the hepatic artery, whereas portal circulation mainly supplies normal hepatic tissues (26). HAIC increases drug concentration in local lesions, prolongs drug action time, directly leads to tumor cell death, and inhibits tumor proliferation. In addition to HAIC, there are other minimally invasive treatment methods, such as transcatheter arterial chemoembolization (TACE), radiofrequency ablation (RFA), etc. But compared to HAIC, TACE mainly focuses on embolism, leading to more severe liver function damage, and affecting treatment compliance (27). HAIC is effective for both detectable liver lesions and intrahepatic micrometastases, whereas the therapeutic efficacy of RFA was reduced for large tumors, and the presence of as many as four or five lesions was considered suitable. HAIC has been used to increase the local drug concentration of liver metastases, thereby increasing liver disease control and the resectability of colorectal cancer with liver metastasis (28). The efficacy of HAIC in gastric cancer with liver metastasis is still uncertain.

Most of the HAI data on liver metastases from gastric cancer involve Asian patients. The largest analysis was a phase II study that included 88 patients, 55 of whom had not received any prior chemotherapy. It evaluates regimen 5-FU/epirubicin/mitomycin C for HAI administration. In 63 evaluable patients, the response rate was 55.6%. Toxicity was mild, with an incidence of 30% nausea -vomiting (2.5%, grade 3-4) and so on (29).

Qiang et al. analyzed 21 patients with gastric cancer with extensive liver metastases who were treated with oral S-1 and HAI oxaliplatin plus floxuridine (FUDR), the overall response rate was 76.2% (9.5% complete response). Intrahepatic and extrahepatic median progression-free survival times were 9.5 and 5.2 months, respectively. MST was 12.3 months. None of the patients experienced grade 4 adverse effects. Grade 3 toxic effects included bone marrow suppression (14.3%) and diarrhea (9.5%) (30). A study of Western patients showed that seven patients received HAIC with a median duration of six cycles. The treatment was feasible and safe, and no grade 3-4 adverse effects had been observed. One patient had stable liver metastases within 7 months (29).

Ojima et al. reported one case that survived for more than 5 years without any signs of recurrence after treatment with HAIC (20). Toyokawa et al. reported that a patient with gastric cancer with liver metastases was disease-free after more than 12 years of HAIC without further chemotherapy (31). Although the sample size is small, it also provides some references for clinical practice.

We report a case of HAIC combined with trastuzumab in a patient with HER2-positive gastric cancer with liver metastases. The patient was 66 years old, but with jaundice due to extensive liver metastases, his general condition was not good. His ECOG score was 2. Jatoi et al. analyzed 367 patients with gastrointestinal tumors, and a total of 154 patients (42%) were aged ≥ 65 years old. The result showed that patients who were ≥65 years old had worse performance scores (32). For this patient, Trastuzumab plus intravenous chemotherapy is the effective option, but it may not adequately control local symptoms, and toxicity tends to be high. We chose HAIC combined with trastuzumab therapy, the patient’s jaundice subsided, symptoms improved, tumor markers decreased, and tumors shrank significantly. The patient’s ECOG score returned to a level of 1. The patient achieved disease control with an intensive regimen followed by less toxic maintenance therapy to delay disease progression.

The choice of maintenance therapy should be based on the patient’s age, physical condition, concomitant diseases, previous treatment, patient preference, economic status, clinical practice bias, and drug accessibility. Li et al. published a prospective observational study that compared maintenance with trastuzumab alone versus the combination of trastuzumab plus mono-chemo-agent (capecitabine or S1) derived from the initial chemotherapy. There were no significant differences in OS (16.5 vs 20.0 months, HR 0.71 P = 0.169) and PFS (7.9 vs 11.0 months, HR 1.06, P = 0.892) between the two groups, although the addition of a chemo-agent to trastuzumab led to a 29% reduction in mortality risk. The safety profile was also similar for both arms (33).

After 6 cycles of intensive therapy, the patient’s liver metastases were locally controlled. He benefited from this therapy. According to the different studies available (33, 34), we recommend the combination of trastuzumab and capecitabine for maintenance treatment to delay disease progression. Trastuzumab combined with capecitabine maintenance therapy followed up for more than 16 months. With only one patient, the limitations of the analysis were obvious. Firstly, since there is only one case, we cannot cover all adverse events. Secondly, the patient’s follow-up time is still short, and the results require long-term follow-up and validation. Thirdly, this protocol did not routinely use the combination of HAIC and trastuzumab, suggesting that efficacy evaluation must be further objectified and standardized through prospective multicenter clinical trials.

In conclusion, HAIC may be an effective treatment, and it may be a traditional tolerable treatment option for patients with major liver metastases from cancer who do not tolerate more intense chemotherapy regimens. This case indicates that HAIC combined with trastuzumab may be a safe and effective treatment selection for extensive GCLM who refused or cannot tolerate first-line intravenous chemotherapy, and may achieve a high-local response and help prolong patient survival.

## Data availability statement

The original contributions presented in the study are included in the article/supplementary material. Further inquiries can be directed to the corresponding author.

## Ethics statement

The studies involving humans were approved by Zhengzhou Third People’s Hospital Ethics Review Committee. The studies were conducted in accordance with the local legislation and institutional requirements. Written informed consent for participation was not required from the participants or the participants’ legal guardians/next of kin in accordance with the national legislation and institutional requirements. Written informed consent was obtained from the individual(s) for the publication of any potentially identifiable images or data included in this article.

## Author contributions

HL: Writing – original draft, Data curation. QW: Formal Analysis, Writing – review & editing. LZ: Investigation, Writing – review & editing. JL: Project administration, Writing – review & editing. YW: Methodology, Writing – review & editing. LW: Resources, Writing – review & editing. LY: Writing – review & editing, Supervision.
